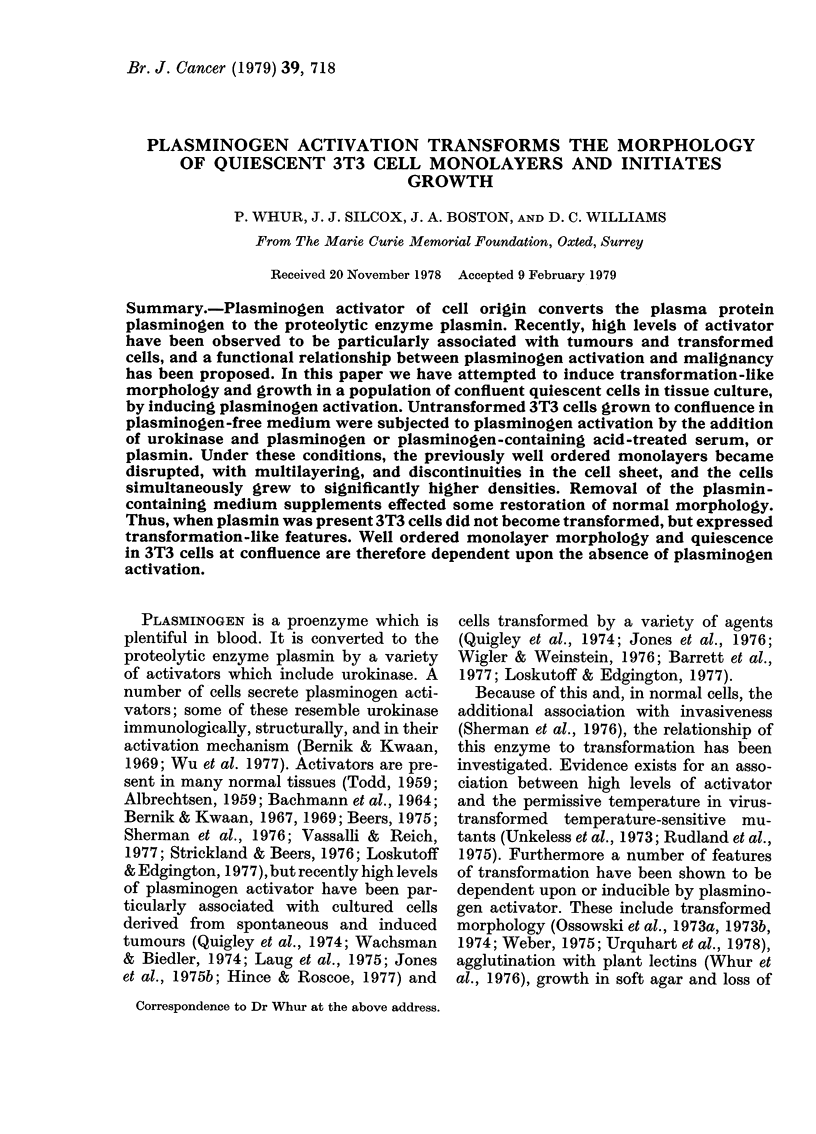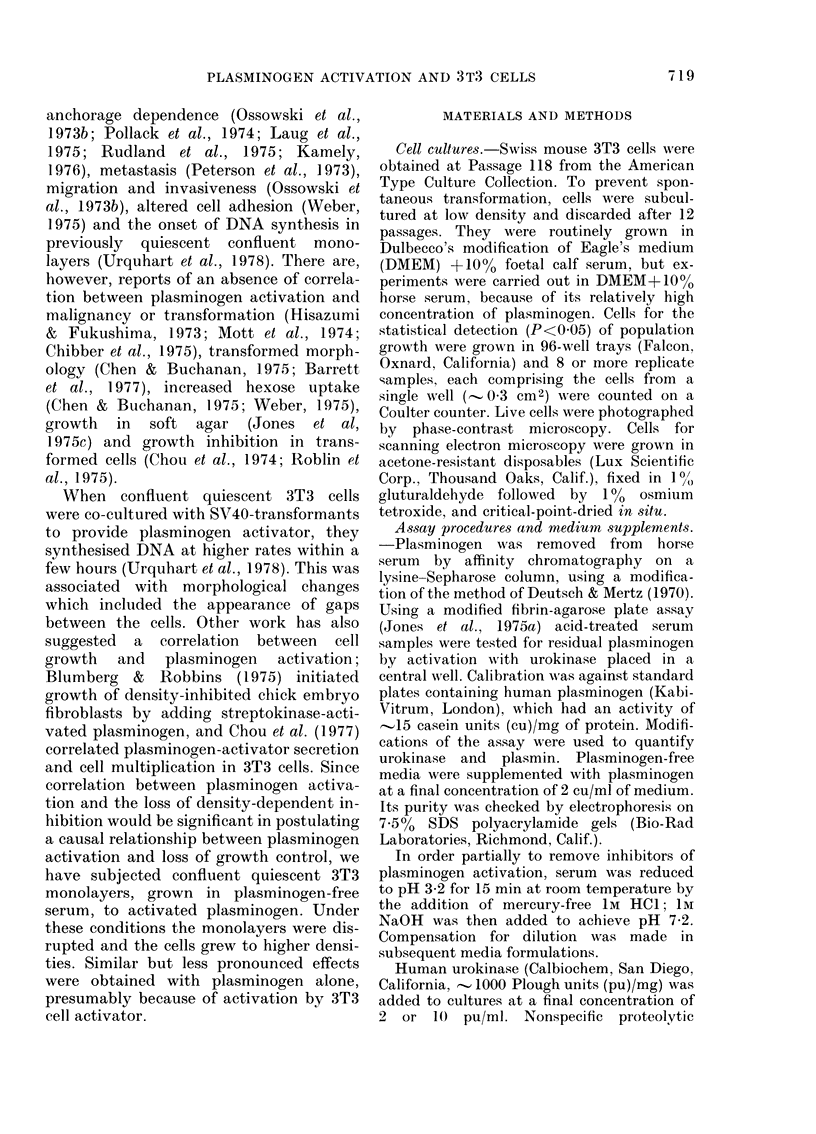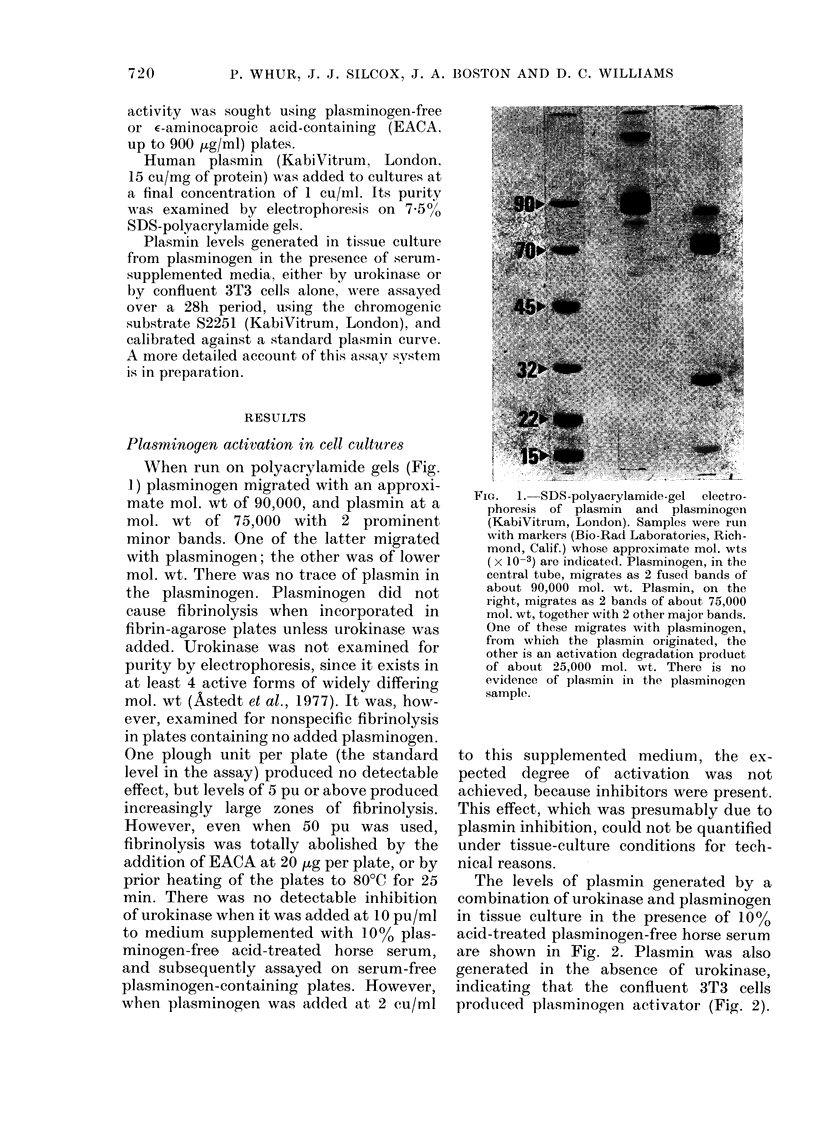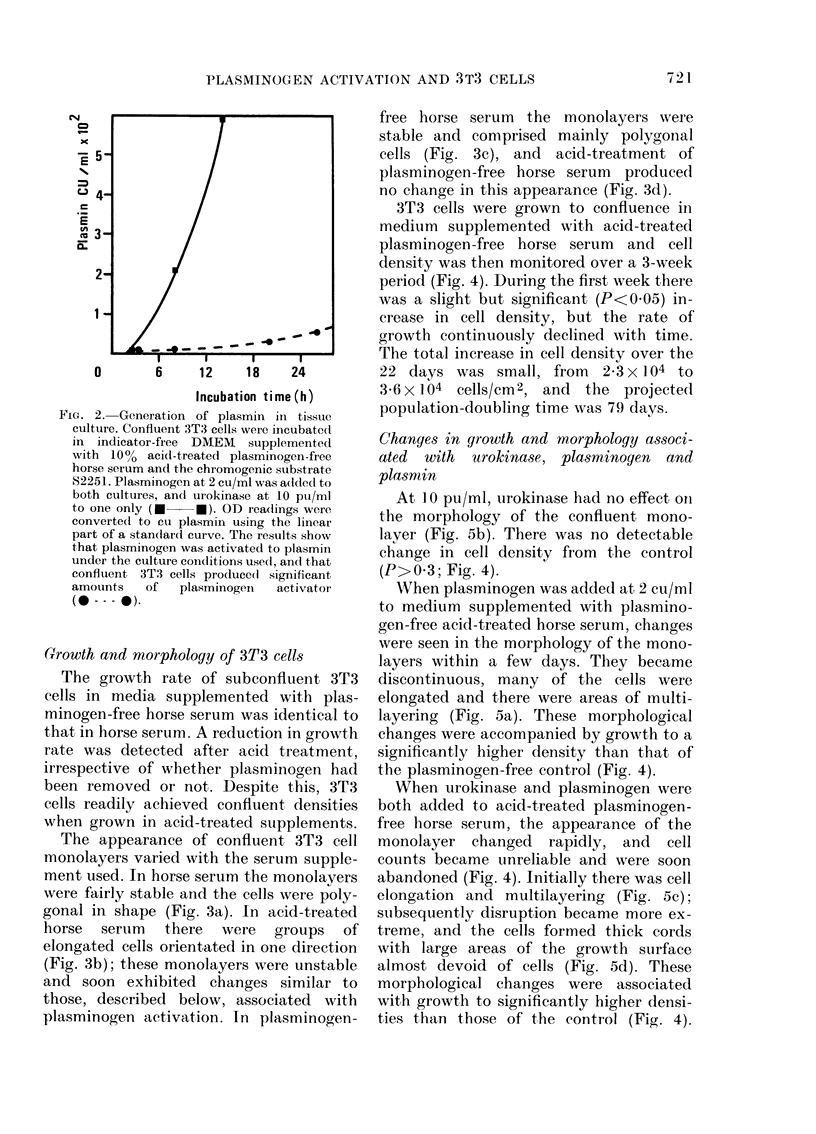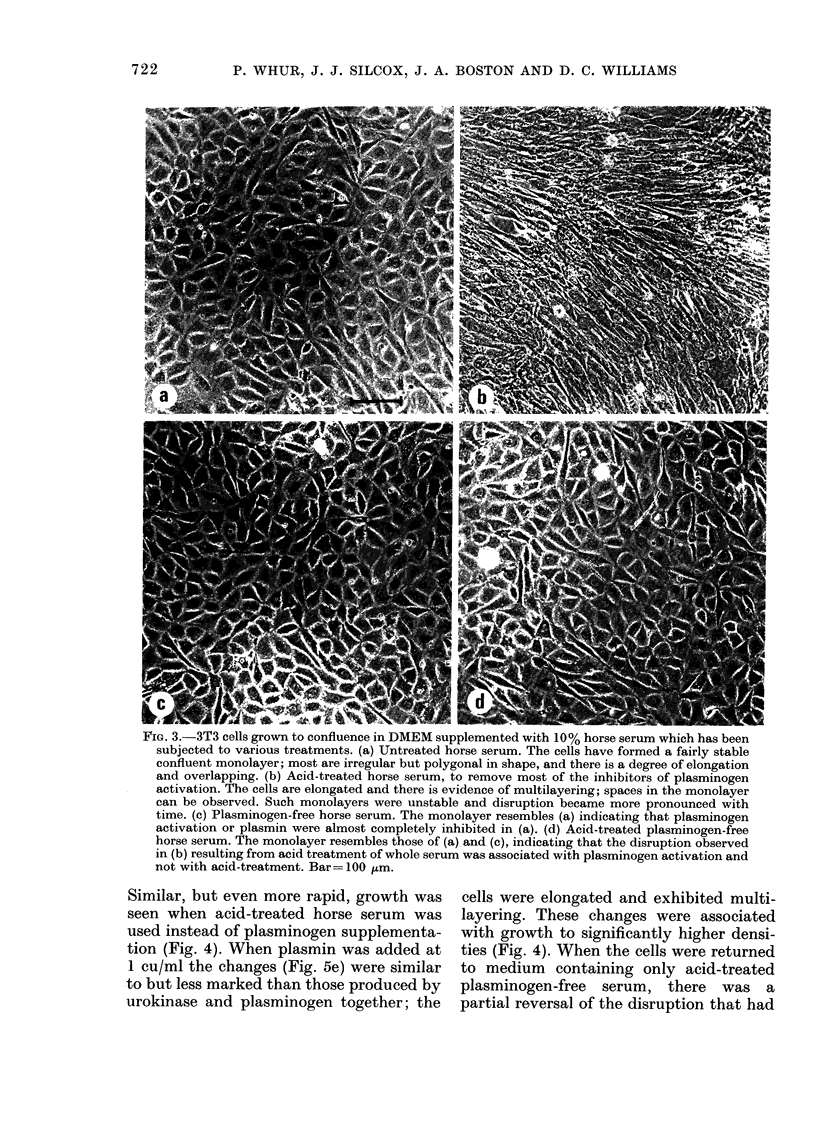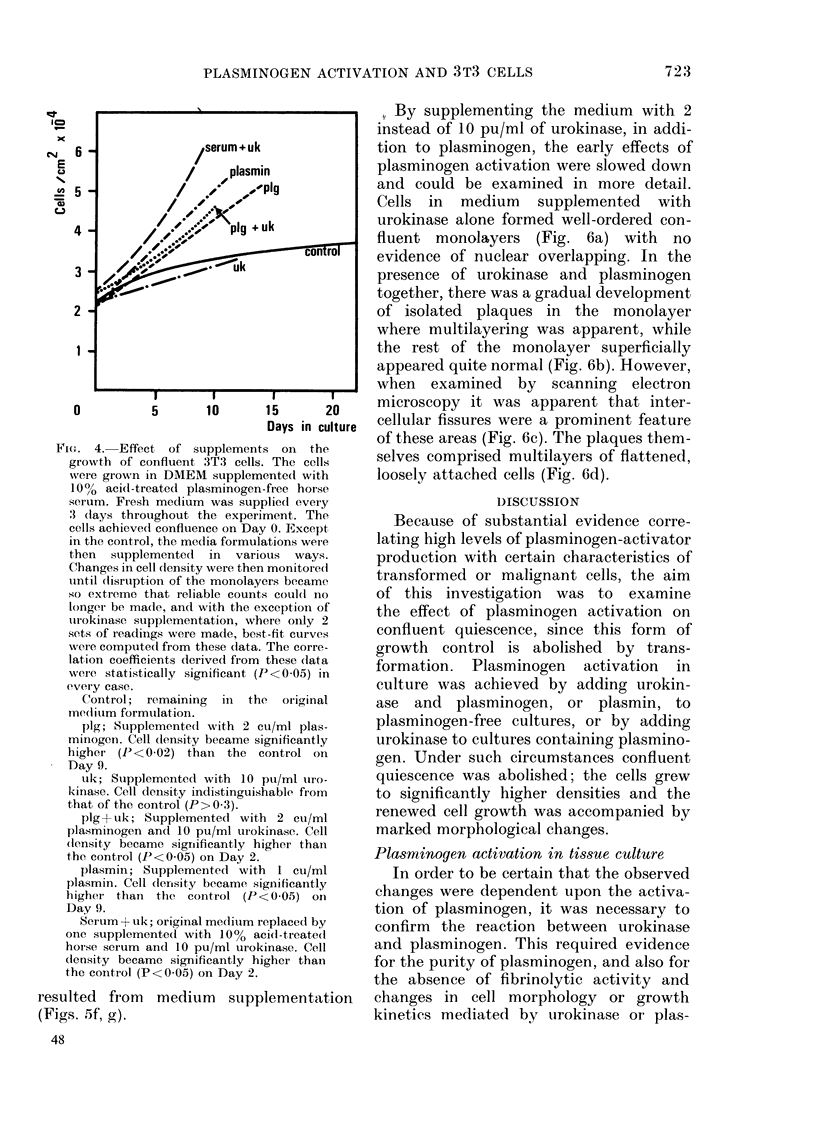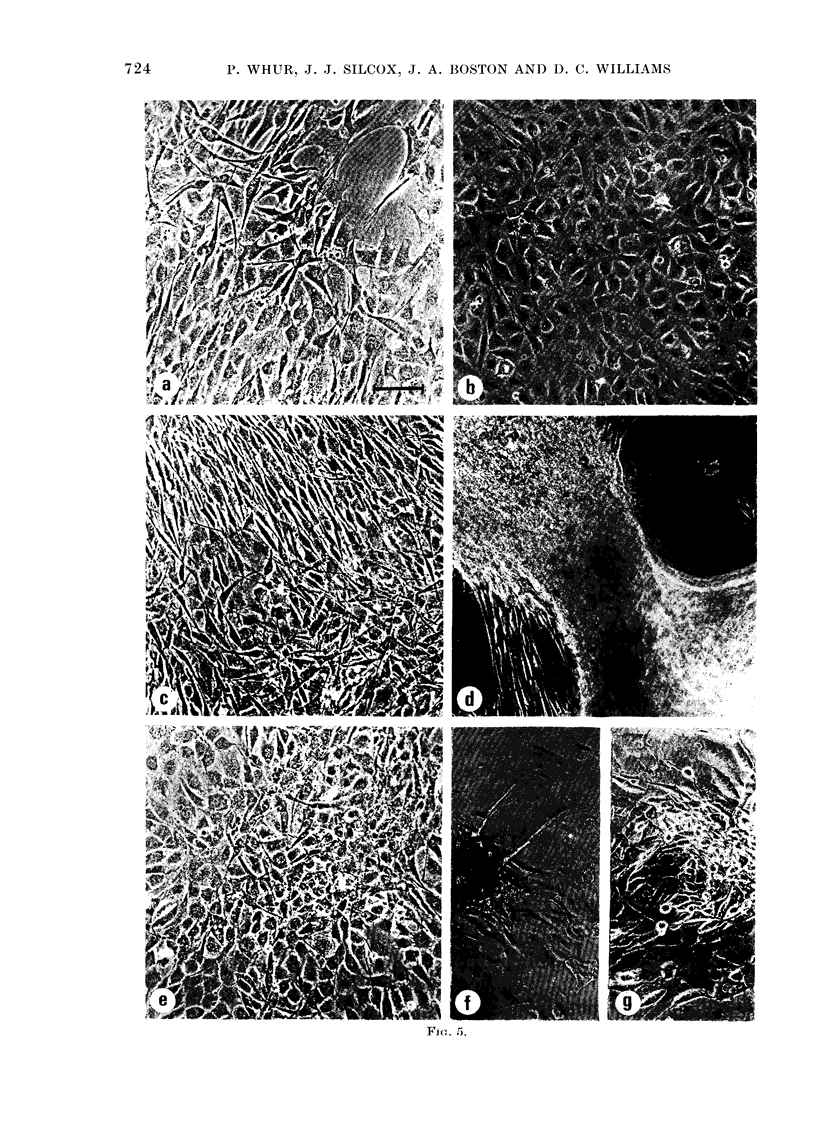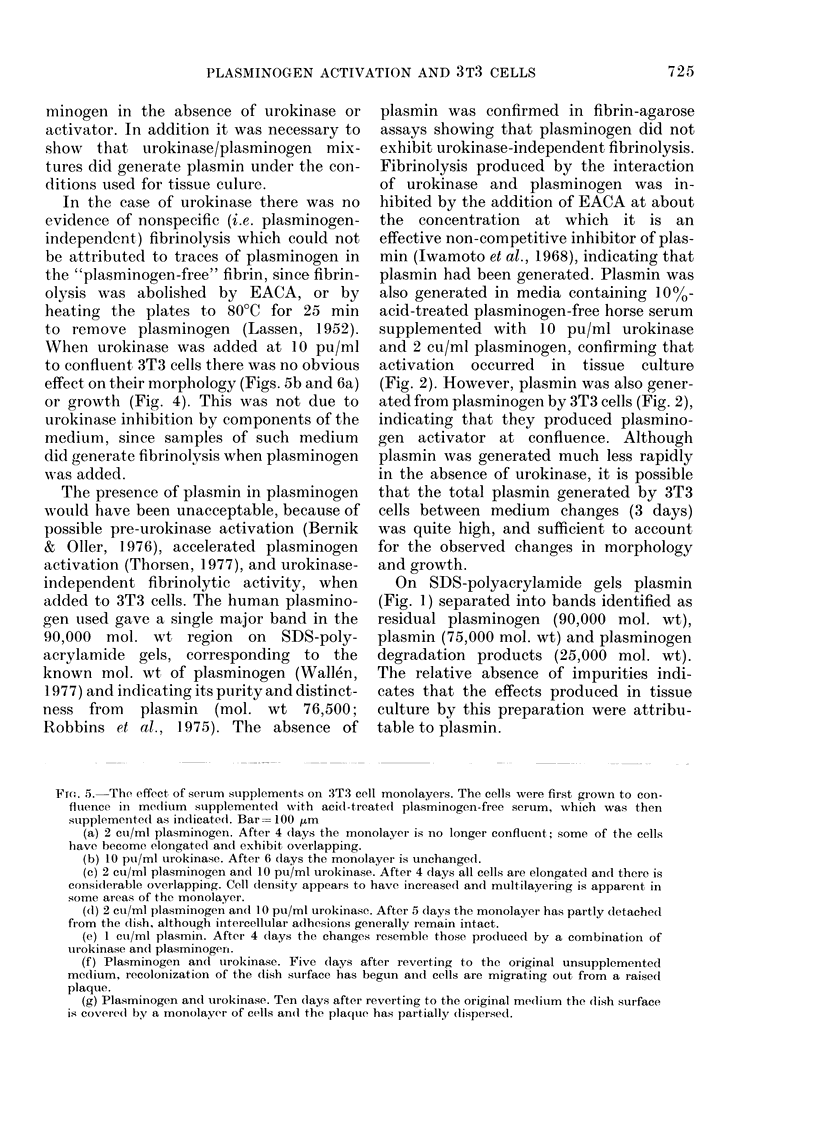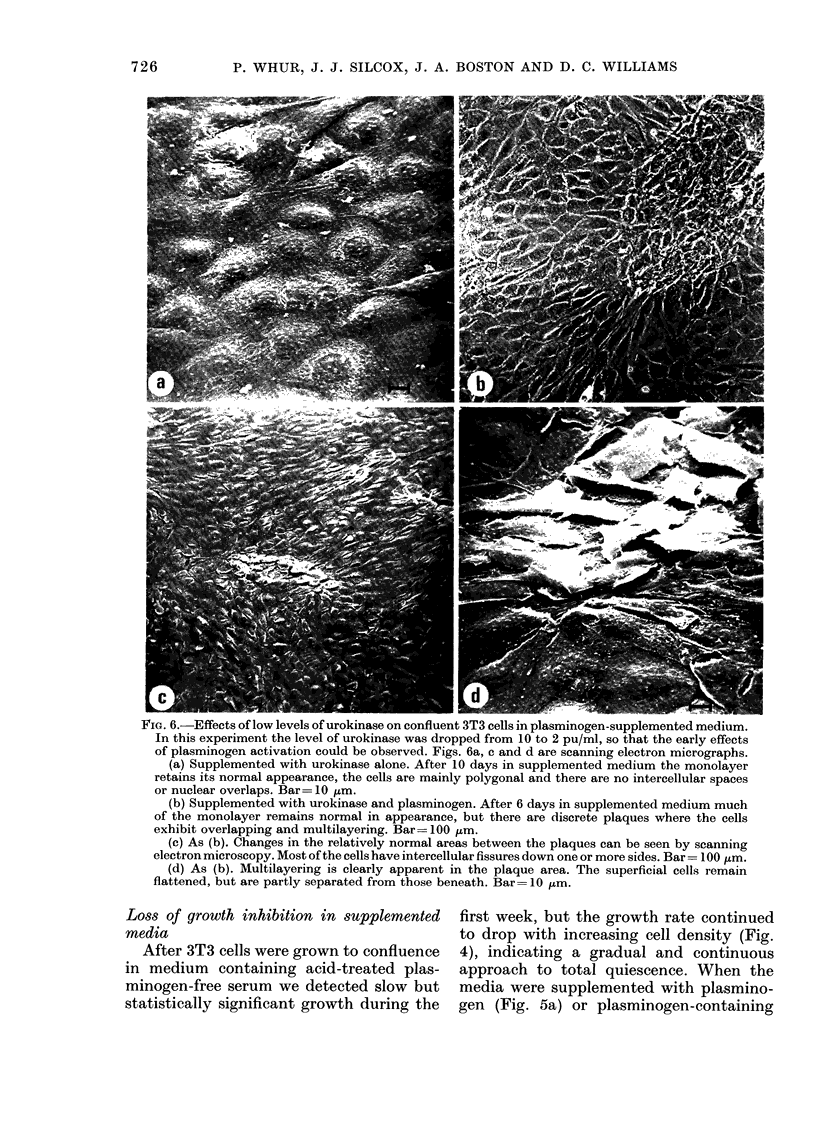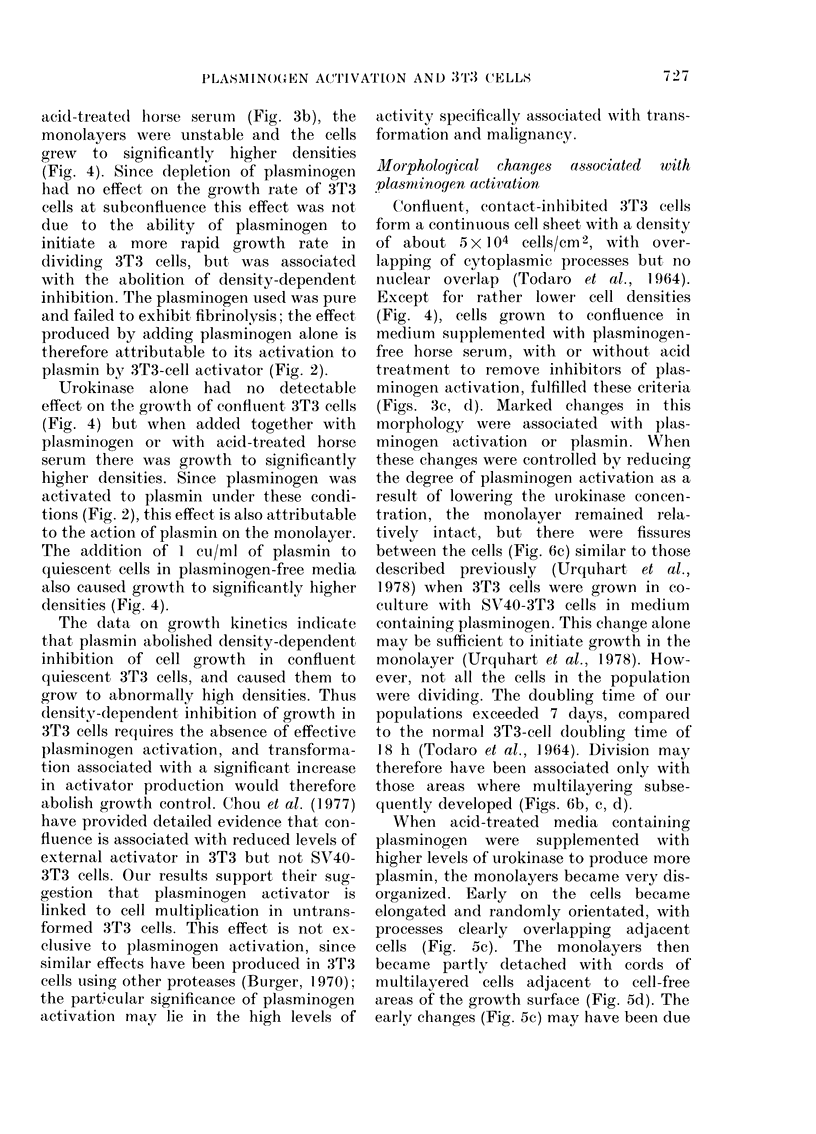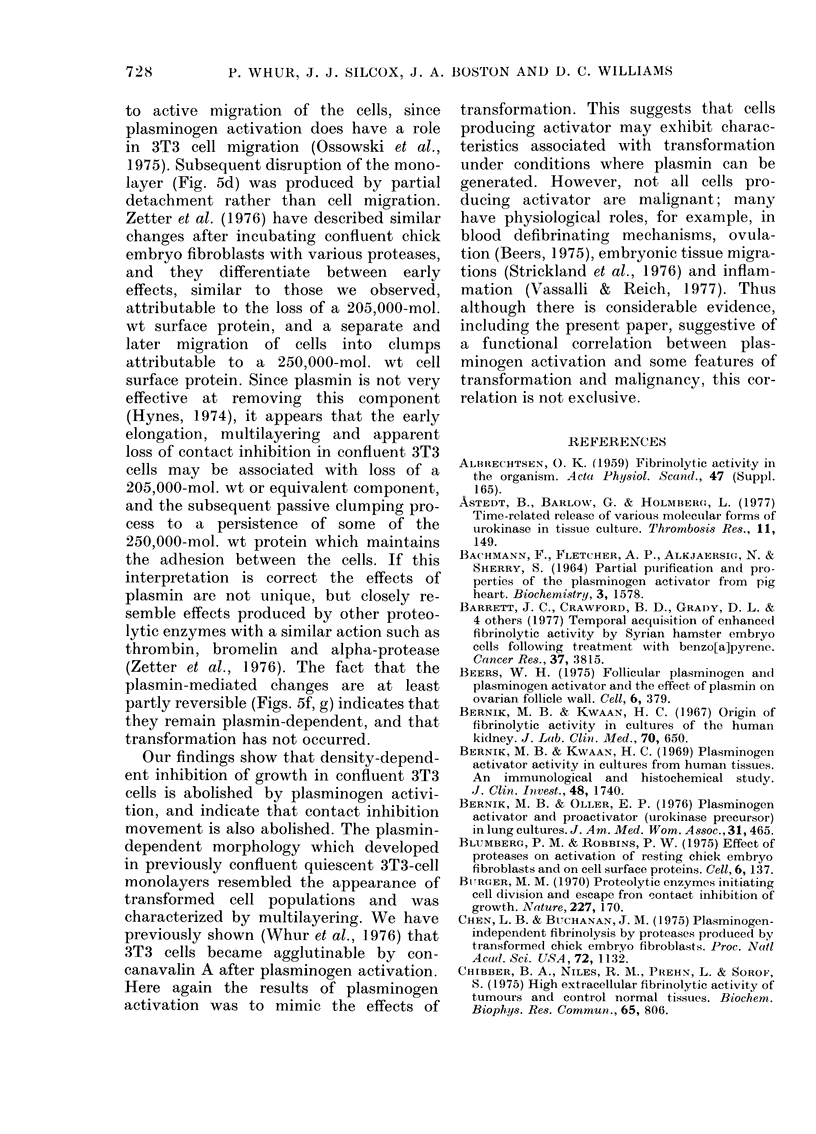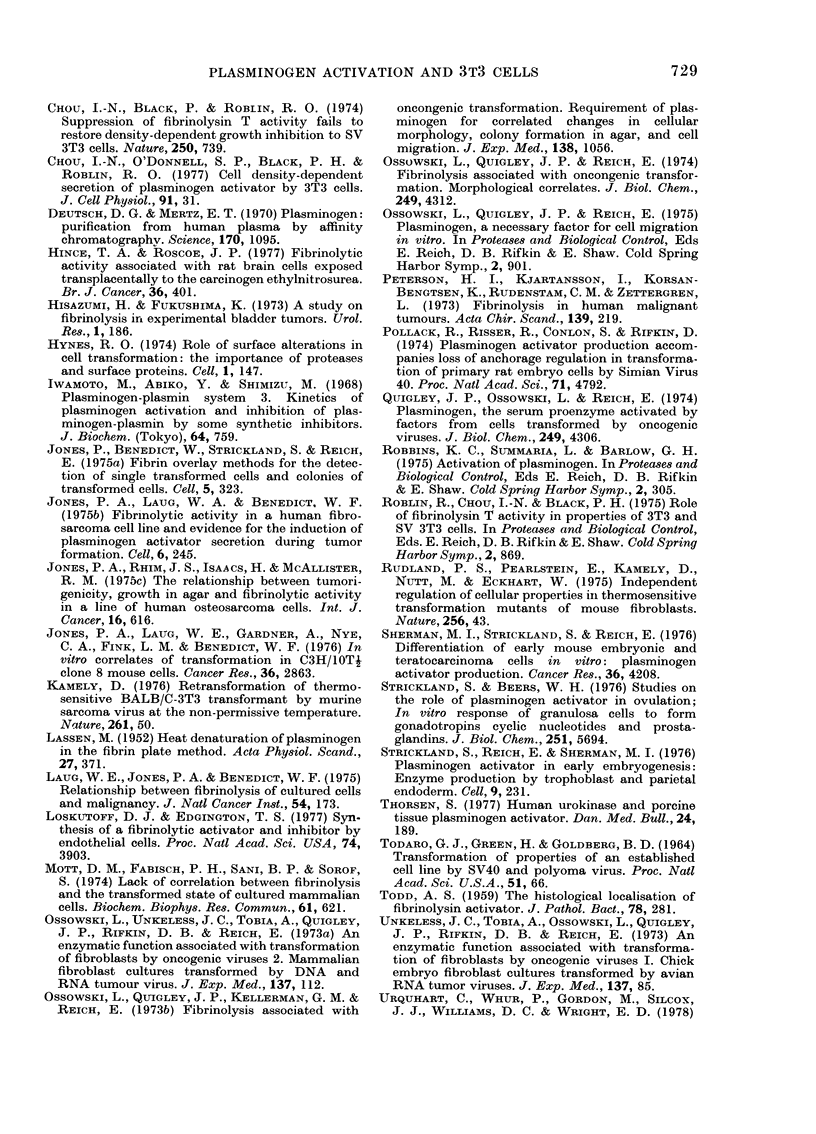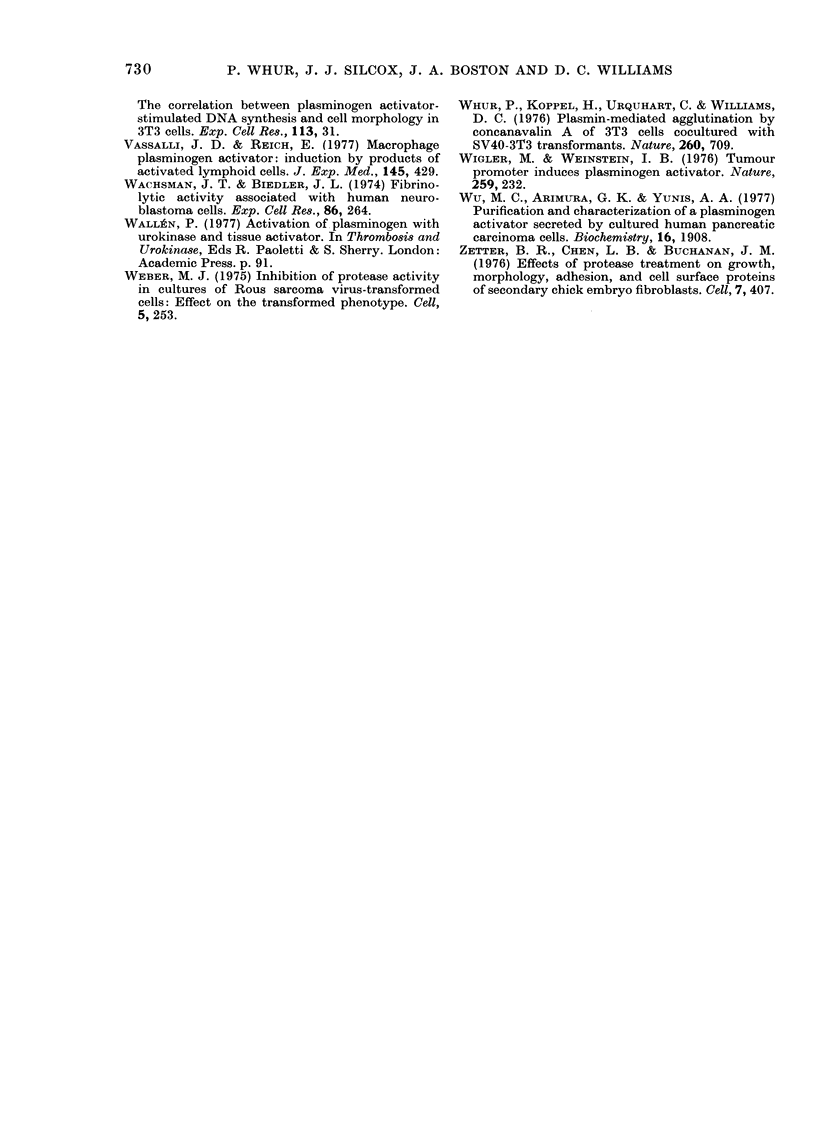# Plasminogen activation transforms the morphology of quiescent 3T3 cell monolayers and initiates growth.

**DOI:** 10.1038/bjc.1979.126

**Published:** 1979-06

**Authors:** P. Whur, J. J. Silcox, J. A. Boston, D. C. Williams

## Abstract

**Images:**


*Br. J. Cancer* (1979) **39**, 718

### PLASMINOGEN ACTIVATION TRANSFORMS THE MORPHOLOGY OF QUIESCENT 3T3 CELL MONOLAYERS AND INITIATES GROWTH

P. WHUR, J. J. SILCOX, J. A. BOSTON, AND D. C. WILLIAMS

*From The Marie Curie Memorial Foundation, Oxted, Surrey*

Received 20 November 1978 Accepted 9 February 1979

**Summary.—Plasminogen activator of cell origin converts the plasma protein plasminogen to the proteolytic enzyme plasmin. Recently, high levels of activator have been observed to be particularly associated with tumours and transformed cells, and a functional relationship between plasminogen activation and malignancy has been proposed. In this paper we have attempted to induce transformation-like morphology and growth in a population of confluent quiescent cells in tissue culture, by inducing plasminogen activation. Untransformed 3T3 cells grown to confluence in plasminogen-free medium were subjected to plasminogen activation by the addition of urokinase and plasminogen or plasminogen-containing acid-treated serum, or plasmin. Under these conditions, the previously well ordered monolayers became disrupted, with multilayering, and discontinuities in the cell sheet, and the cells simultaneously grew to significantly higher densities. Removal of the plasmin-containing medium supplements effected some restoration of normal morphology. Thus, when plasmin was present 3T3 cells did not become transformed, but expressed transformation-like features. Well ordered monolayer morphology and quiescence in 3T3 cells at confluence are therefore dependent upon the absence of plasminogen activation.**

Plasminogen is a proenzyme which is plentiful in blood. It is converted to the proteolytic enzyme plasmin by a variety of activators which include urokinase. A number of cells secrete plasminogen activators; some of these resemble urokinase immunologically, structurally, and in their activation mechanism (Bernik & Kwaan, 1969; Wu *et al.* 1977). Activators are present in many normal tissues (Todd, 1959; Albrechtsen, 1959; Bachmann *et al.*, 1964; Bernik & Kwaan, 1967, 1969; Beers, 1975; Sherman *et al.*, 1976; Vassalli & Reich, 1977; Strickland & Beers, 1976; Loskutoff & Edgington, 1977), but recently high levels of plasminogen activator have been particularly associated with cultured cells derived from spontaneous and induced tumours (Quigley *et al.*, 1974; Wachsman & Biedler, 1974; Laug *et al.*, 1975; Jones *et al.*, 1975*b*; Hince & Roscoe, 1977) and cells transformed by a variety of agents (Quigley *et al.*, 1974; Jones *et al.*, 1976; Wigler & Weinstein, 1976; Barrett *et al.*, 1977; Loskutoff & Edgington, 1977).

Because of this and, in normal cells, the additional association with invasiveness (Sherman *et al.*, 1976), the relationship of this enzyme to transformation has been investigated. Evidence exists for an association between high levels of activator and the permissive temperature in virus-transformed temperature-sensitive mutants (Unkeless *et al.*, 1973; Rudland *et al.*, 1975). Furthermore a number of features of transformation have been shown to be dependent upon or inducible by plasminogen activator. These include transformed morphology (Ossowski *et al.*, 1973*a*, 1973*b*, 1974; Weber, 1975; Urquhart *et al.*, 1978), agglutination with plant lectins (Whur *et al.*, 1976), growth in soft agar and loss of

Correspondence to Dr Whur at the above address.

anchorage dependence (Ossowski *et al.*, 1973*b*; Pollack *et al.*, 1974; Laug *et al.*, 1975; Rudland *et al.*, 1975; Kamely, 1976), metastasis (Peterson *et al.*, 1973), migration and invasiveness (Ossowski *et al.*, 1973*b*), altered cell adhesion (Weber, 1975) and the onset of DNA synthesis in previously quiescent confluent monolayers (Urquhart *et al.*, 1978). There are, however, reports of an absence of correlation between plasminogen activation and malignancy or transformation (Hisazumi & Fukushima, 1973; Mott *et al.*, 1974; Chibber *et al.*, 1975), transformed morphology (Chen & Buchanan, 1975; Barrett *et al.*, 1977), increased hexose uptake (Chen & Buchanan, 1975; Weber, 1975), growth in soft agar (Jones *et al*, 1975*c*) and growth inhibition in transformed cells (Chou *et al.*, 1974; Roblin *et al.*, 1975).

When confluent quiescent 3T3 cells were co-cultured with SV40-transformants to provide plasminogen activator, they synthesised DNA at higher rates within a few hours (Urquhart *et al.*, 1978). This was associated with morphological changes which included the appearance of gaps between the cells. Other work has also suggested a correlation between cell growth and plasminogen activation; Blumberg & Robbins (1975) initiated growth of density-inhibited chick embryo fibroblasts by adding streptokinase-activated plasminogen, and Chou *et al.* (1977) correlated plasminogen-activator secretion and cell multiplication in 3T3 cells. Since correlation between plasminogen activation and the loss of density-dependent inhibition would be significant in postulating a causal relationship between plasminogen activation and loss of growth control, we have subjected confluent quiescent 3T3 monolayers, grown in plasminogen-free serum, to activated plasminogen. Under these conditions the monolayers were disrupted and the cells grew to higher densities. Similar but less pronounced effects were obtained with plasminogen alone, presumably because of activation by 3T3 cell activator.

### MATERIALS AND METHODS

*Cell cultures.*—Swiss mouse 3T3 cells were obtained at Passage 118 from the American Type Culture Collection. To prevent spontaneous transformation, cells were subcultured at low density and discarded after 12 passages. They were routinely grown in Dulbecco's modification of Eagle's medium (DMEM) +10% foetal calf serum, but experiments were carried out in DMEM+10% horse serum, because of its relatively high concentration of plasminogen. Cells for the statistical detection ($P<0{\cdot}05$) of population growth were grown in 96-well trays (Falcon, Oxnard, California) and 8 or more replicate samples, each comprising the cells from a single well ($\sim 0{\cdot}3$ cm$^2$) were counted on a Coulter counter. Live cells were photographed by phase-contrast microscopy. Cells for scanning electron microscopy were grown in acetone-resistant disposables (Lux Scientific Corp., Thousand Oaks, Calif.), fixed in 1% gluturaldehyde followed by 1% osmium tetroxide, and critical-point-dried *in situ*.

*Assay procedures and medium supplements.*—Plasminogen was removed from horse serum by affinity chromatography on a lysine–Sepharose column, using a modification of the method of Deutsch & Mertz (1970). Using a modified fibrin-agarose plate assay (Jones *et al.*, 1975*a*) acid-treated serum samples were tested for residual plasminogen by activation with urokinase placed in a central well. Calibration was against standard plates containing human plasminogen (Kabi-Vitrum, London), which had an activity of $\sim$15 casein units (cu)/mg of protein. Modifications of the assay were used to quantify urokinase and plasmin. Plasminogen-free media were supplemented with plasminogen at a final concentration of 2 cu/ml of medium. Its purity was checked by electrophoresis on 7·5% SDS polyacrylamide gels (Bio-Rad Laboratories, Richmond, Calif.).

In order partially to remove inhibitors of plasminogen activation, serum was reduced to pH 3·2 for 15 min at room temperature by the addition of mercury-free 1M HCl; 1M NaOH was then added to achieve pH 7·2. Compensation for dilution was made in subsequent media formulations.

Human urokinase (Calbiochem, San Diego, California, $\sim$1000 Plough units (pu)/mg) was added to cultures at a final concentration of 2 or 10 pu/ml. Nonspecific proteolytic activity was sought using plasminogen-free or ε-aminocaproic acid-containing (EACA, up to 900 μg/ml) plates.

Human plasmin (KabiVitrum, London, 15 cu/mg of protein) was added to cultures at a final concentration of 1 cu/ml. Its purity was examined by electrophoresis on 7·5% SDS-polyacrylamide gels.

Plasmin levels generated in tissue culture from plasminogen in the presence of serum-supplemented media, either by urokinase or by confluent 3T3 cells alone, were assayed over a 28h period, using the chromogenic substrate S2251 (KabiVitrum, London), and calibrated against a standard plasmin curve. A more detailed account of this assay system is in preparation.

FIG. 1.—SDS-polyacrylamide-gel electrophoresis of plasmin and plasminogen (KabiVitrum, London). Samples were run with markers (Bio-Rad Laboratories, Richmond, Calif.) whose approximate mol. wts ($\times 10^{-3}$) are indicated. Plasminogen, in the central tube, migrates as 2 fused bands of about 90,000 mol. wt. Plasmin, on the right, migrates as 2 bands of about 75,000 mol. wt, together with 2 other major bands. One of these migrates with plasminogen, from which the plasmin originated, the other is an activation degradation product of about 25,000 mol. wt. There is no evidence of plasmin in the plasminogen sample.

### RESULTS

### *Plasminogen activation in cell cultures*

When run on polyacrylamide gels (Fig. 1) plasminogen migrated with an approximate mol. wt of 90,000, and plasmin at a mol. wt of 75,000 with 2 prominent minor bands. One of the latter migrated with plasminogen; the other was of lower mol. wt. There was no trace of plasmin in the plasminogen. Plasminogen did not cause fibrinolysis when incorporated in fibrin-agarose plates unless urokinase was added. Urokinase was not examined for purity by electrophoresis, since it exists in at least 4 active forms of widely differing mol. wt (Åstedt *et al.*, 1977). It was, however, examined for nonspecific fibrinolysis in plates containing no added plasminogen. One plough unit per plate (the standard level in the assay) produced no detectable effect, but levels of 5 pu or above produced increasingly large zones of fibrinolysis. However, even when 50 pu was used, fibrinolysis was totally abolished by the addition of EACA at 20 μg per plate, or by prior heating of the plates to 80°C for 25 min. There was no detectable inhibition of urokinase when it was added at 10 pu/ml to medium supplemented with 10% plasminogen-free acid-treated horse serum, and subsequently assayed on serum-free plasminogen-containing plates. However, when plasminogen was added at 2 cu/ml to this supplemented medium, the expected degree of activation was not achieved, because inhibitors were present. This effect, which was presumably due to plasmin inhibition, could not be quantified under tissue-culture conditions for technical reasons.

The levels of plasmin generated by a combination of urokinase and plasminogen in tissue culture in the presence of 10% acid-treated plasminogen-free horse serum are shown in Fig. 2. Plasmin was also generated in the absence of urokinase, indicating that the confluent 3T3 cells produced plasminogen activator (Fig. 2).

FIG. 2.—Generation of plasmin in tissue culture. Confluent 3T3 cells were incubated in indicator-free DMEM supplemented with 10% acid-treated plasminogen-free horse serum and the chromogenic substrate S2251. Plasminogen at 2 cu/ml was added to both cultures, and urokinase at 10 pu/ml to one only (■——■). OD readings were converted to cu plasmin using the linear part of a standard curve. The results show that plasminogen was activated to plasmin under the culture conditions used, and that confluent 3T3 cells produced significant amounts of plasminogen activator (● - - - ●).

*Growth and morphology of 3T3 cells*

The growth rate of subconfluent 3T3 cells in media supplemented with plasminogen-free horse serum was identical to that in horse serum. A reduction in growth rate was detected after acid treatment, irrespective of whether plasminogen had been removed or not. Despite this, 3T3 cells readily achieved confluent densities when grown in acid-treated supplements.

The appearance of confluent 3T3 cell monolayers varied with the serum supplement used. In horse serum the monolayers were fairly stable and the cells were polygonal in shape (Fig. 3a). In acid-treated horse serum there were groups of elongated cells orientated in one direction (Fig. 3b); these monolayers were unstable and soon exhibited changes similar to those, described below, associated with plasminogen activation. In plasminogen-free horse serum the monolayers were stable and comprised mainly polygonal cells (Fig. 3c), and acid-treatment of plasminogen-free horse serum produced no change in this appearance (Fig. 3d).

3T3 cells were grown to confluence in medium supplemented with acid-treated plasminogen-free horse serum and cell density was then monitored over a 3-week period (Fig. 4). During the first week there was a slight but significant ($P<0{\cdot}05$) increase in cell density, but the rate of growth continuously declined with time. The total increase in cell density over the 22 days was small, from $2{\cdot}3\times10^4$ to $3{\cdot}6\times10^4$ cells/cm$^2$, and the projected population-doubling time was 79 days.

*Changes in growth and morphology associated with urokinase, plasminogen and plasmin*

At 10 pu/ml, urokinase had no effect on the morphology of the confluent monolayer (Fig. 5b). There was no detectable change in cell density from the control ($P>0{\cdot}3$; Fig. 4).

When plasminogen was added at 2 cu/ml to medium supplemented with plasminogen-free acid-treated horse serum, changes were seen in the morphology of the monolayers within a few days. They became discontinuous, many of the cells were elongated and there were areas of multilayering (Fig. 5a). These morphological changes were accompanied by growth to a significantly higher density than that of the plasminogen-free control (Fig. 4).

When urokinase and plasminogen were both added to acid-treated plasminogen-free horse serum, the appearance of the monolayer changed rapidly, and cell counts became unreliable and were soon abandoned (Fig. 4). Initially there was cell elongation and multilayering (Fig. 5c); subsequently disruption became more extreme, and the cells formed thick cords with large areas of the growth surface almost devoid of cells (Fig. 5d). These morphological changes were associated with growth to significantly higher densities than those of the control (Fig. 4).

FIG. 3.—3T3 cells grown to confluence in DMEM supplemented with 10% horse serum which has been subjected to various treatments. (a) Untreated horse serum. The cells have formed a fairly stable confluent monolayer; most are irregular but polygonal in shape, and there is a degree of elongation and overlapping. (b) Acid-treated horse serum, to remove most of the inhibitors of plasminogen activation. The cells are elongated and there is evidence of multilayering; spaces in the monolayer can be observed. Such monolayers were unstable and disruption became more pronounced with time. (c) Plasminogen-free horse serum. The monolayer resembles (a) indicating that plasminogen activation or plasmin were almost completely inhibited in (a). (d) Acid-treated plasminogen-free horse serum. The monolayer resembles those of (a) and (c), indicating that the disruption observed in (b) resulting from acid treatment of whole serum was associated with plasminogen activation and not with acid-treatment. Bar = 100 $\mu$m.

Similar, but even more rapid, growth was seen when acid-treated horse serum was used instead of plasminogen supplementation (Fig. 4). When plasmin was added at 1 cu/ml the changes (Fig. 5e) were similar to but less marked than those produced by urokinase and plasminogen together; the cells were elongated and exhibited multilayering. These changes were associated with growth to significantly higher densities (Fig. 4). When the cells were returned to medium containing only acid-treated plasminogen-free serum, there was a partial reversal of the disruption that had

FIG. 4.—Effect of supplements on the growth of confluent 3T3 cells. The cells were grown in DMEM supplemented with 10% acid-treated plasminogen-free horse serum. Fresh medium was supplied every 3 days throughout the experiment. The cells achieved confluence on Day 0. Except in the control, the media formulations were then supplemented in various ways. Changes in cell density were then monitored until disruption of the monolayers became so extreme that reliable counts could no longer be made, and with the exception of urokinase supplementation, where only 2 sets of readings were made, best-fit curves were computed from these data. The correlation coefficients derived from these data were statistically significant ($P<0\cdot05$) in every case.

Control; remaining in the original medium formulation.

plg; Supplemented with 2 cu/ml plasminogen. Cell density became significantly higher ($P<0\cdot02$) than the control on Day 9.

uk; Supplemented with 10 pu/ml urokinase. Cell density indistinguishable from that of the control ($P>0\cdot3$).

plg+uk; Supplemented with 2 cu/ml plasminogen and 10 pu/ml urokinase. Cell density became significantly higher than the control ($P<0\cdot05$) on Day 2.

plasmin; Supplemented with 1 cu/ml plasmin. Cell density became significantly higher than the control ($P<0\cdot05$) on Day 9.

Serum+uk; original medium replaced by one supplemented with 10% acid-treated horse serum and 10 pu/ml urokinase. Cell density became significantly higher than the control ($P<0\cdot05$) on Day 2.

resulted from medium supplementation (Figs. 5f, g).

By supplementing the medium with 2 instead of 10 pu/ml of urokinase, in addition to plasminogen, the early effects of plasminogen activation were slowed down and could be examined in more detail. Cells in medium supplemented with urokinase alone formed well-ordered confluent monolayers (Fig. 6a) with no evidence of nuclear overlapping. In the presence of urokinase and plasminogen together, there was a gradual development of isolated plaques in the monolayer where multilayering was apparent, while the rest of the monolayer superficially appeared quite normal (Fig. 6b). However, when examined by scanning electron microscopy it was apparent that intercellular fissures were a prominent feature of these areas (Fig. 6c). The plaques themselves comprised multilayers of flattened, loosely attached cells (Fig. 6d).

### DISCUSSION

Because of substantial evidence correlating high levels of plasminogen-activator production with certain characteristics of transformed or malignant cells, the aim of this investigation was to examine the effect of plasminogen activation on confluent quiescence, since this form of growth control is abolished by transformation. Plasminogen activation in culture was achieved by adding urokinase and plasminogen, or plasmin, to plasminogen-free cultures, or by adding urokinase to cultures containing plasminogen. Under such circumstances confluent quiescence was abolished; the cells grew to significantly higher densities and the renewed cell growth was accompanied by marked morphological changes.

### *Plasminogen activation in tissue culture*

In order to be certain that the observed changes were dependent upon the activation of plasminogen, it was necessary to confirm the reaction between urokinase and plasminogen. This required evidence for the purity of plasminogen, and also for the absence of fibrinolytic activity and changes in cell morphology or growth kinetics mediated by urokinase or plas-

FIG. 5.

minogen in the absence of urokinase or activator. In addition it was necessary to show that urokinase/plasminogen mixtures did generate plasmin under the conditions used for tissue culure.

In the case of urokinase there was no evidence of nonspecific (*i.e.* plasminogen-independent) fibrinolysis which could not be attributed to traces of plasminogen in the "plasminogen-free" fibrin, since fibrinolysis was abolished by EACA, or by heating the plates to 80°C for 25 min to remove plasminogen (Lassen, 1952). When urokinase was added at 10 pu/ml to confluent 3T3 cells there was no obvious effect on their morphology (Figs. 5b and 6a) or growth (Fig. 4). This was not due to urokinase inhibition by components of the medium, since samples of such medium did generate fibrinolysis when plasminogen was added.

The presence of plasmin in plasminogen would have been unacceptable, because of possible pre-urokinase activation (Bernik & Oller, 1976), accelerated plasminogen activation (Thorsen, 1977), and urokinase-independent fibrinolytic activity, when added to 3T3 cells. The human plasminogen used gave a single major band in the 90,000 mol. wt region on SDS-polyacrylamide gels, corresponding to the known mol. wt of plasminogen (Wallén, 1977) and indicating its purity and distinctness from plasmin (mol. wt 76,500; Robbins *et al.*, 1975). The absence of plasmin was confirmed in fibrin-agarose assays showing that plasminogen did not exhibit urokinase-independent fibrinolysis. Fibrinolysis produced by the interaction of urokinase and plasminogen was inhibited by the addition of EACA at about the concentration at which it is an effective non-competitive inhibitor of plasmin (Iwamoto *et al.*, 1968), indicating that plasmin had been generated. Plasmin was also generated in media containing 10%-acid-treated plasminogen-free horse serum supplemented with 10 pu/ml urokinase and 2 cu/ml plasminogen, confirming that activation occurred in tissue culture (Fig. 2). However, plasmin was also generated from plasminogen by 3T3 cells (Fig. 2), indicating that they produced plasminogen activator at confluence. Although plasmin was generated much less rapidly in the absence of urokinase, it is possible that the total plasmin generated by 3T3 cells between medium changes (3 days) was quite high, and sufficient to account for the observed changes in morphology and growth.

On SDS-polyacrylamide gels plasmin (Fig. 1) separated into bands identified as residual plasminogen (90,000 mol. wt), plasmin (75,000 mol. wt) and plasminogen degradation products (25,000 mol. wt). The relative absence of impurities indicates that the effects produced in tissue culture by this preparation were attributable to plasmin.

FIG. 5.—The effect of serum supplements on 3T3 cell monolayers. The cells were first grown to confluence in medium supplemented with acid-treated plasminogen-free serum, which was then supplemented as indicated. Bar = 100 μm

(a) 2 cu/ml plasminogen. After 4 days the monolayer is no longer confluent; some of the cells have become elongated and exhibit overlapping.

(b) 10 pu/ml urokinase. After 6 days the monolayer is unchanged.

(c) 2 cu/ml plasminogen and 10 pu/ml urokinase. After 4 days all cells are elongated and there is considerable overlapping. Cell density appears to have increased and multilayering is apparent in some areas of the monolayer.

(d) 2 cu/ml plasminogen and 10 pu/ml urokinase. After 5 days the monolayer has partly detached from the dish, although intercellular adhesions generally remain intact.

(e) 1 cu/ml plasmin. After 4 days the changes resemble those produced by a combination of urokinase and plasminogen.

(f) Plasminogen and urokinase. Five days after reverting to the original unsupplemented medium, recolonization of the dish surface has begun and cells are migrating out from a raised plaque.

(g) Plasminogen and urokinase. Ten days after reverting to the original medium the dish surface is covered by a monolayer of cells and the plaque has partially dispersed.

FIG. 6.—Effects of low levels of urokinase on confluent 3T3 cells in plasminogen-supplemented medium. In this experiment the level of urokinase was dropped from 10 to 2 pu/ml, so that the early effects of plasminogen activation could be observed. Figs. 6a, c and d are scanning electron micrographs.
(a) Supplemented with urokinase alone. After 10 days in supplemented medium the monolayer retains its normal appearance, the cells are mainly polygonal and there are no intercellular spaces or nuclear overlaps. Bar = 10 $\mu$m.
(b) Supplemented with urokinase and plasminogen. After 6 days in supplemented medium much of the monolayer remains normal in appearance, but there are discrete plaques where the cells exhibit overlapping and multilayering. Bar = 100 $\mu$m.
(c) As (b). Changes in the relatively normal areas between the plaques can be seen by scanning electron microscopy. Most of the cells have intercellular fissures down one or more sides. Bar = 100 $\mu$m.
(d) As (b). Multilayering is clearly apparent in the plaque area. The superficial cells remain flattened, but are partly separated from those beneath. Bar = 10 $\mu$m.

*Loss of growth inhibition in supplemented media*

After 3T3 cells were grown to confluence in medium containing acid-treated plasminogen-free serum we detected slow but statistically significant growth during the first week, but the growth rate continued to drop with increasing cell density (Fig. 4), indicating a gradual and continuous approach to total quiescence. When the media were supplemented with plasminogen (Fig. 5a) or plasminogen-containing acid-treated horse serum (Fig. 3b), the monolayers were unstable and the cells grew to significantly higher densities (Fig. 4). Since depletion of plasminogen had no effect on the growth rate of 3T3 cells at subconfluence this effect was not due to the ability of plasminogen to initiate a more rapid growth rate in dividing 3T3 cells, but was associated with the abolition of density-dependent inhibition. The plasminogen used was pure and failed to exhibit fibrinolysis; the effect produced by adding plasminogen alone is therefore attributable to its activation to plasmin by 3T3-cell activator (Fig. 2).

Urokinase alone had no detectable effect on the growth of confluent 3T3 cells (Fig. 4) but when added together with plasminogen or with acid-treated horse serum there was growth to significantly higher densities. Since plasminogen was activated to plasmin under these conditions (Fig. 2), this effect is also attributable to the action of plasmin on the monolayer. The addition of 1 cu/ml of plasmin to quiescent cells in plasminogen-free media also caused growth to significantly higher densities (Fig. 4).

The data on growth kinetics indicate that plasmin abolished density-dependent inhibition of cell growth in confluent quiescent 3T3 cells, and caused them to grow to abnormally high densities. Thus density-dependent inhibition of growth in 3T3 cells requires the absence of effective plasminogen activation, and transformation associated with a significant increase in activator production would therefore abolish growth control. Chou *et al.* (1977) have provided detailed evidence that confluence is associated with reduced levels of external activator in 3T3 but not SV40-3T3 cells. Our results support their suggestion that plasminogen activator is linked to cell multiplication in untransformed 3T3 cells. This effect is not exclusive to plasminogen activation, since similar effects have been produced in 3T3 cells using other proteases (Burger, 1970); the particular significance of plasminogen activation may lie in the high levels of activity specifically associated with transformation and malignancy.

*Morphological changes associated with plasminogen activation*

Confluent, contact-inhibited 3T3 cells form a continuous cell sheet with a density of about $5 \times 10^4$ cells/cm$^2$, with overlapping of cytoplasmic processes but no nuclear overlap (Todaro *et al.*, 1964). Except for rather lower cell densities (Fig. 4), cells grown to confluence in medium supplemented with plasminogen-free horse serum, with or without acid treatment to remove inhibitors of plasminogen activation, fulfilled these criteria (Figs. 3c, d). Marked changes in this morphology were associated with plasminogen activation or plasmin. When these changes were controlled by reducing the degree of plasminogen activation as a result of lowering the urokinase concentration, the monolayer remained relatively intact, but there were fissures between the cells (Fig. 6c) similar to those described previously (Urquhart *et al.*, 1978) when 3T3 cells were grown in co-culture with SV40-3T3 cells in medium containing plasminogen. This change alone may be sufficient to initiate growth in the monolayer (Urquhart *et al.*, 1978). However, not all the cells in the population were dividing. The doubling time of our populations exceeded 7 days, compared to the normal 3T3-cell doubling time of 18 h (Todaro *et al.*, 1964). Division may therefore have been associated only with those areas where multilayering subsequently developed (Figs. 6b, c, d).

When acid-treated media containing plasminogen were supplemented with higher levels of urokinase to produce more plasmin, the monolayers became very disorganized. Early on the cells became elongated and randomly orientated, with processes clearly overlapping adjacent cells (Fig. 5c). The monolayers then became partly detached with cords of multilayered cells adjacent to cell-free areas of the growth surface (Fig. 5d). The early changes (Fig. 5c) may have been due to active migration of the cells, since plasminogen activation does have a role in 3T3 cell migration (Ossowski *et al.*, 1975). Subsequent disruption of the monolayer (Fig. 5d) was produced by partial detachment rather than cell migration. Zetter *et al.* (1976) have described similar changes after incubating confluent chick embryo fibroblasts with various proteases, and they differentiate between early effects, similar to those we observed, attributable to the loss of a 205,000-mol. wt surface protein, and a separate and later migration of cells into clumps attributable to a 250,000-mol. wt cell surface protein. Since plasmin is not very effective at removing this component (Hynes, 1974), it appears that the early elongation, multilayering and apparent loss of contact inhibition in confluent 3T3 cells may be associated with loss of a 205,000-mol. wt or equivalent component, and the subsequent passive clumping process to a persistence of some of the 250,000-mol. wt protein which maintains the adhesion between the cells. If this interpretation is correct the effects of plasmin are not unique, but closely resemble effects produced by other proteolytic enzymes with a similar action such as thrombin, bromelin and alpha-protease (Zetter *et al.*, 1976). The fact that the plasmin-mediated changes are at least partly reversible (Figs. 5f, g) indicates that they remain plasmin-dependent, and that transformation has not occurred.

Our findings show that density-dependent inhibition of growth in confluent 3T3 cells is abolished by plasminogen activition, and indicate that contact inhibition movement is also abolished. The plasmin-dependent morphology which developed in previously confluent quiescent 3T3-cell monolayers resembled the appearance of transformed cell populations and was characterized by multilayering. We have previously shown (Whur *et al.*, 1976) that 3T3 cells became agglutinable by concanavalin A after plasminogen activation. Here again the results of plasminogen activation was to mimic the effects of transformation. This suggests that cells producing activator may exhibit characteristics associated with transformation under conditions where plasmin can be generated. However, not all cells producing activator are malignant; many have physiological roles, for example, in blood defibrinating mechanisms, ovulation (Beers, 1975), embryonic tissue migrations (Strickland *et al.*, 1976) and inflammation (Vassalli & Reich, 1977). Thus although there is considerable evidence, including the present paper, suggestive of a functional correlation between plasminogen activation and some features of transformation and malignancy, this correlation is not exclusive.

### REFERENCES

ALBRECHTSEN, O. K. (1959) Fibrinolytic activity in the organism. *Acta Physiol. Scand.*, **47** (Suppl. 165).

ÅSTEDT, B., BARLOW, G. & HOLMBERG, L. (1977) Time-related release of various molecular forms of urokinase in tissue culture. *Thrombosis Res.*, **11**, 149.

BACHMANN, F., FLETCHER, A. P., ALKJAERSIG, N. & SHERRY, S. (1964) Partial purification and properties of the plasminogen activator from pig heart. *Biochemistry*, **3**, 1578.

BARRETT, J. C., CRAWFORD, B. D., GRADY, D. L. & 4 others (1977) Temporal acquisition of enhanced fibrinolytic activity by Syrian hamster embryo cells following treatment with benzo[a]pyrene. *Cancer Res.*, **37**, 3815.

BEERS, W. H. (1975) Follicular plasminogen and plasminogen activator and the effect of plasmin on ovarian follicle wall. *Cell*, **6**, 379.

BERNIK, M. B. & KWAAN, H. C. (1967) Origin of fibrinolytic activity in cultures of the human kidney. *J. Lab. Clin. Med.*, **70**, 650.

BERNIK, M. B. & KWAAN, H. C. (1969) Plasminogen activator activity in cultures from human tissues. An immunological and histochemical study. *J. Clin. Invest.*, **48**, 1740.

BERNIK, M. B. & OLLER, E. P. (1976) Plasminogen activator and proactivator (urokinase precursor) in lung cultures. *J. Am. Med. Wom. Assoc.*, **31**, 465.

BLUMBERG, P. M. & ROBBINS, P. W. (1975) Effect of proteases on activation of resting chick embryo fibroblasts and on cell surface proteins. *Cell*, **6**, 137.

BURGER, M. M. (1970) Proteolytic enzymes initiating cell division and escape fron contact inhibition of growth. *Nature*, **227**, 170.

CHEN, L. B. & BUCHANAN, J. M. (1975) Plasminogen-independent fibrinolysis by proteases produced by transformed chick embryo fibroblasts. *Proc. Natl Acad. Sci. USA*, **72**, 1132.

CHIBBER, B. A., NILES, R. M., PREHN, L. & SOROF, S. (1975) High extracellular fibrinolytic activity of tumours and control normal tissues. *Biochem. Biophys. Res. Commun.*, **65**, 806.

CHOU, I.-N., BLACK, P. & ROBLIN, R. O. (1974) Suppression of fibrinolysin T activity fails to restore density-dependent growth inhibition to SV 3T3 cells. *Nature*, **250**, 739.

CHOU, I.-N., O'DONNELL, S. P., BLACK, P. H. & ROBLIN, R. O. (1977) Cell density-dependent secretion of plasminogen activator by 3T3 cells. *J. Cell Physiol.*, **91**, 31.

DEUTSCH, D. G. & MERTZ, E. T. (1970) Plasminogen: purification from human plasma by affinity chromatography. *Science*, **170**, 1095.

HINCE, T. A. & ROSCOE, J. P. (1977) Fibrinolytic activity associated with rat brain cells exposed transplacentally to the carcinogen ethylnitrosurea. *Br. J. Cancer*, **36**, 401.

HISAZUMI, H. & FUKUSHIMA, K. (1973) A study on fibrinolysis in experimental bladder tumors. *Urol. Res.*, **1**, 186.

HYNES, R. O. (1974) Role of surface alterations in cell transformation: the importance of proteases and surface proteins. *Cell*, **1**, 147.

IWAMOTO, M., ABIKO, Y. & SHIMIZU, M. (1968) Plasminogen-plasmin system 3. Kinetics of plasminogen activation and inhibition of plasminogen-plasmin by some synthetic inhibitors. *J. Biochem.* (Tokyo), **64**, 759.

JONES, P., BENEDICT, W., STRICKLAND, S. & REICH, E. (1975*a*) Fibrin overlay methods for the detection of single transformed cells and colonies of transformed cells. *Cell*, **5**, 323.

JONES, P. A., LAUG, W. A. & BENEDICT, W. F. (1975*b*) Fibrinolytic activity in a human fibrosarcoma cell line and evidence for the induction of plasminogen activator secretion during tumor formation. *Cell*, **6**, 245.

JONES, P. A., RHIM, J. S., ISAACS, H. & MCALLISTER, R. M. (1975*c*) The relationship between tumorigenicity, growth in agar and fibrinolytic activity in a line of human osteosarcoma cells. *Int. J. Cancer*, **16**, 616.

JONES, P. A., LAUG, W. E., GARDNER, A., NYE, C. A., FINK, L. M. & BENEDICT, W. F. (1976) *In vitro* correlates of transformation in C3H/10T½ clone 8 mouse cells. *Cancer Res.*, **36**, 2863.

KAMELY, D. (1976) Retransformation of thermosensitive BALB/C-3T3 transformant by murine sarcoma virus at the non-permissive temperature. *Nature*, **261**, 50.

LASSEN, M. (1952) Heat denaturation of plasminogen in the fibrin plate method. *Acta Physiol. Scand.*, **27**, 371.

LAUG, W. E., JONES, P. A. & BENEDICT, W. F. (1975) Relationship between fibrinolysis of cultured cells and malignancy. *J. Natl Cancer Inst.*, **54**, 173.

LOSKUTOFF, D. J. & EDGINGTON, T. S. (1977) Synthesis of a fibrinolytic activator and inhibitor by endothelial cells. *Proc. Natl Acad. Sci. USA*, **74**, 3903.

MOTT, D. M., FABISCH, P. H., SANI, B. P. & SOROF, S. (1974) Lack of correlation between fibrinolysis and the transformed state of cultured mammalian cells. *Biochem. Biophys. Res. Commun.*, **61**, 621.

OSSOWSKI, L., UNKELESS, J. C., TOBIA, A., QUIGLEY, J. P., RIFKIN, D. B. & REICH, E. (1973*a*) An enzymatic function associated with transformation of fibroblasts by oncogenic viruses 2. Mammalian fibroblast cultures transformed by DNA and RNA tumour virus. *J. Exp. Med.*, **137**, 112.

OSSOWSKI, L., QUIGLEY, J. P., KELLERMAN, G. M. & REICH, E. (1973*b*) Fibrinolysis associated with oncongenic transformation. Requirement of plasminogen for correlated changes in cellular morphology, colony formation in agar, and cell migration. *J. Exp. Med.*, **138**, 1056.

OSSOWSKI, L., QUIGLEY, J. P. & REICH, E. (1974) Fibrinolysis associated with oncongenic transformation. Morphological correlates. *J. Biol. Chem.*, **249**, 4312.

OSSOWSKI, L., QUIGLEY, J. P. & REICH, E. (1975) Plasminogen, a necessary factor for cell migration *in vitro*. In *Proteases and Biological Control*, Eds E. Reich, D. B. Rifkin & E. Shaw. Cold Spring Harbor Symp., **2**, 901.

PETERSON, H. I., KJARTANSSON, I., KORSAN-BENGTSEN, K., RUDENSTAM, C. M. & ZETTERGREN, L. (1973) Fibrinolysis in human malignant tumours. *Acta Chir. Scand.*, **139**, 219.

POLLACK, R., RISSER, R., CONLON, S. & RIFKIN, D. (1974) Plasminogen activator production accompanies loss of anchorage regulation in transformation of primary rat embryo cells by Simian Virus 40. *Proc. Natl Acad. Sci.*, **71**, 4792.

QUIGLEY, J. P., OSSOWSKI, L. & REICH, E. (1974) Plasminogen, the serum proenzyme activated by factors from cells transformed by oncogenic viruses. *J. Biol. Chem.*, **249**, 4306.

ROBBINS, K. C., SUMMARIA, L. & BARLOW, G. H. (1975) Activation of plasminogen. In *Proteases and Biological Control*, Eds E. Reich, D. B. Rifkin & E. Shaw. *Cold Spring Harbor Symp.*, **2**, 305.

ROBLIN, R., CHOU, I.-N. & BLACK, P. H. (1975) Role of fibrinolysin T activity in properties of 3T3 and SV 3T3 cells. In *Proteases and Biological Control*, Eds. E. Reich, D. B. Rifkin & E. Shaw. *Cold Spring Harbor Symp.*, **2**, 869.

RUDLAND, P. S., PEARLSTEIN, E., KAMELY, D., NUTT, M. & ECKHART, W. (1975) Independent regulation of cellular properties in thermosensitive transformation mutants of mouse fibroblasts. *Nature*, **256**, 43.

SHERMAN, M. I., STRICKLAND, S. & REICH, E. (1976) Differentiation of early mouse embryonic and teratocarcinoma cells *in vitro*: plasminogen activator production. *Cancer Res.*, **36**, 4208.

STRICKLAND, S. & BEERS, W. H. (1976) Studies on the role of plasminogen activator in ovulation; *In vitro* response of granulosa cells to form gonadotropins cyclic nucleotides and prostaglandins. *J. Biol. Chem.*, **251**, 5694.

STRICKLAND, S., REICH, E. & SHERMAN, M. I. (1976) Plasminogen activator in early embryogenesis: Enzyme production by trophoblast and parietal endoderm. *Cell*, **9**, 231.

THORSEN, S. (1977) Human urokinase and porcine tissue plasminogen activator. *Dan. Med. Bull.*, **24**, 189.

TODARO, G. J., GREEN, H. & GOLDBERG, B. D. (1964) Transformation of properties of an established cell line by SV40 and polyoma virus. *Proc. Natl Acad. Sci. U.S.A.*, **51**, 66.

TODD, A. S. (1959) The histological localisation of fibrinolysin activator. *J. Pathol. Bact.*, **78**, 281.

UNKELESS, J. C., TOBIA, A., OSSOWSKI, L., QUIGLEY, J. P., RIFKIN, D. B. & REICH, E. (1973) An enzymatic function associated with transformation of fibroblasts by oncogenic viruses I. Chick embryo fibroblast cultures transformed by avian RNA tumor viruses. *J. Exp. Med.*, **137**, 85.

URQUHART, C., WHUR, P., GORDON, M., SILCOX, J. J., WILLIAMS, D. C. & WRIGHT, E. D. (1978)

The correlation between plasminogen activator-stimulated DNA synthesis and cell morphology in 3T3 cells. *Exp. Cell Res.*, **113**, 31.

VASSALLI, J. D. & REICH, E. (1977) Macrophage plasminogen activator: induction by products of activated lymphoid cells. *J. Exp. Med.*, **145**, 429.

WACHSMAN, J. T. & BIEDLER, J. L. (1974) Fibrinolytic activity associated with human neuroblastoma cells. *Exp. Cell Res.*, **86**, 264.

WALLÉN, P. (1977) Activation of plasminogen with urokinase and tissue activator. In *Thrombosis and Urokinase*, Eds R. Paoletti & S. Sherry. London: Academic Press. p. 91.

WEBER, M. J. (1975) Inhibition of protease activity in cultures of Rous sarcoma virus-transformed cells: Effect on the transformed phenotype. *Cell*, **5**, 253.

WHUR, P., KOPPEL, H., URQUHART, C. & WILLIAMS, D. C. (1976) Plasmin-mediated agglutination by concanavalin A of 3T3 cells cocultured with SV40-3T3 transformants. *Nature*, **260**, 709.

WIGLER, M. & WEINSTEIN, I. B. (1976) Tumour promoter induces plasminogen activator. *Nature*, **259**, 232.

WU, M. C., ARIMURA, G. K. & YUNIS, A. A. (1977) Purification and characterization of a plasminogen activator secreted by cultured human pancreatic carcinoma cells. *Biochemistry*, **16**, 1908.

ZETTER, B. R., CHEN, L. B. & BUCHANAN, J. M. (1976) Effects of protease treatment on growth, morphology, adhesion, and cell surface proteins of secondary chick embryo fibroblasts. *Cell*, **7**, 407.